# Health Care Utilization for Common Shoulder Disorders: Analysis of the 2010–2019 National Patient Sample Data from the Health Insurance Review and Assessment Service in Korea

**DOI:** 10.3390/medicina60050744

**Published:** 2024-04-29

**Authors:** Jin-Young Kang, Doori Kim, Huijun Kim, In-Hyuk Ha, Yoon Jae Lee

**Affiliations:** 1 Jaseng Hospital of Korean Medicine, Seoul 06110, Republic of Korea; jyriver1@jaseng.co.kr; 2 Jaseng Spine and Joint Research Institute, Jaseng Medical Foundation, Seoul 06110, Republic of Koreahanihata@gmail.com (I.-H.H.)

**Keywords:** rotator cuff tear and syndrome, impingement syndrome, adhesive capsulitis, HIRA claims data, national patient sample, cost of care, surgical/non-surgical service utilization

## Abstract

*Background and Objective:* The aim of this study was to analyze trends in surgical and non-surgical service utilization for common shoulder disorders in Korea from 2010 to 2019. *Methods and Materials:* This retrospective, cross-sectional, descriptive study utilized National Patient Sample data from the Health Insurance and Review Assessment Service (HIRA) of Korea. These data constitute a 2% sample out of the entire Korean population and include data for a variety of parameters instrumental for health care research. Patients with at least one medical service use for rotator cuff syndrome or tear, impingement syndrome, or adhesive capsulitis between January 2010 and December 2019 were included. Trends in healthcare utilization by disorder type, patient demographics, seasonal service use, and treatment details were examined. *Results:* There was an upward trend in the total number of patients and costs for shoulder disorders, from 35,798 patients and USD 5,485,196 in 2010 to 42,558 and USD 11,522,543 in 2019, respectively. The number of patients aged ≥60 and hospital visits increased. March had the highest number of claims. Physical therapy was the most common non-surgical procedure, while nerve block claims more than doubled. Opioid prescription rates also tripled. Surgical treatments were dominated by shoulder rotator cuff repair and acromioplasty. *Conclusions:* There was a significant increase in healthcare utilization for shoulder disorders, marked by rising costs and patient numbers. The use of nerve blocks and opioids notably increased. These data are valuable for clinicians, researchers, and policymakers.

## 1. Introduction

The shoulder joint is a highly mobile joint that allows a wide range of movement. The glenohumeral joint of the shoulder has the highest range of motion in the human body [1]. However, this wide range of motion also makes the shoulder joint unstable, leading to a diverse spectrum of pathologies compared with other joints [2]. Shoulder pain from shoulder disorders is a frequently reported musculoskeletal pain, following low back pain. Owing to the inherent instability in the anatomical structure of the shoulder joint and the high frequency of use of these muscles, the number of patients and medical costs related to shoulder disorders has continued to increase [3,4]. Also, the shoulder pathology psychologically afflicts the patient and drastically reduces the patient’s quality of life [5]. Major causes of shoulder pain affecting middle-aged and older individuals include rotator cuff syndrome or tear (RCST), impingement syndrome (IS), and adhesive capsulitis (AC) [3,4,6].

Rotator cuff disorder manifests in a group of four distinct muscles in the shoulder. IS is a condition that occurs when the bones, muscles, tendons, ligaments, and bursae constituting the shoulder are squeezed and rubbed against one another, causing pain and impacting everyday activities [7]. AC involves adhesions across the glenohumeral joint, including the synovium and joint capsule; the condition causes severe pain and reduced range of motion in the affected shoulder [8]. Although these common shoulder disorders have different pathophysiology to some extent, all cause persistent pain and show slow recovery progress, thus undermining the quality of life of the patients, reducing their productivity, and leading to further social and economic burdens.

A systematic review reported an increasing prevalence of rotator cuff disorder with increasing age, from 9.7% for under 20 years of age to 62% for over 80 years of age [9]. One report showed that one in five people in their lifetime presented with shoulder pain complaints and received treatment [10]. In the US, the number of patients with shoulder disorders is increasing, along with the number of shoulder replacement surgeries (total shoulder arthroplasty) [9]. In the UK, shoulder pain is also common, accounting for 7% to 26% of musculoskeletal disorders [11]. The number of patients with shoulder disorders is also increasing in the Korean population [12]. Approximately 2.3 million Koreans are diagnosed with shoulder disorders annually, making it the second most common orthopedic disorder after knee disorders in the country [13]. With the increasing number of patients, the medical costs of shoulder disorders are also increasing, along with the number of days of hospital visits by patients [14].

Regarding the treatment modalities of shoulder disorders, different clinical strategies are applied according to the conditions of individual patients. Notably, clinicians mainly choose analgesics or physical therapy, and for severe pain, intramuscular injections of steroids are used for pain relief [15,16]. In the case of physical therapy, heat/cold therapy or electrical stimulation, such as transcutaneous electrical nerve stimulation and interferential current therapy, is a common method of treatment, as well as exercise therapy [16]. If there is no improvement through non-surgical treatment or in the case of indications for surgery, such as a full-thickness tear of the rotator cuff, surgical treatment is performed, such as repair of the shoulder rotator cuff or acromioplasty. According to recent studies, arthroscopic surgery, which does not involve a large incision as in the case of traditional open surgery, is increasingly preferred [17]. The trend in the treatment of shoulder disorders is also similar in Korea, and a variety of injection therapies are used, such as prolotherapy for injecting small amounts of concentrated glucose [18], polydeoxyribonucleotide or DNA injection [19], and the use of injectable collagen [20]. Treatment modalities of traditional Korean medicine (KM), including acupuncture, pharmacopuncture, and Chuna manual therapy, are also available and popular in Korea.

The increase in the prevalence and medical costs of common shoulder disorders and the variety of treatment modalities available warrant an in-depth investigation of the recent trend of healthcare utilization for the treatment of shoulder disorders. A study examined the trends of shoulder disorders in Korea, but it lacked an analysis of the different types of shoulder disorders and different treatment methods [21]. Researchers have also introduced a range of treatments for shoulder disorders [22,23], but they have discussed only specific modes of treatment, such as arthroscopic surgery [24] or postoperative rehabilitation [25]. Few studies have considered the overall trend of healthcare utilization, such as epidemiology related to the use of medical services for the patient population of shoulder disorders, seasonal trends, or the use of medications. Regarding KM therapy for shoulder disorders, studies have examined the aspect of effectiveness, taking into account the dual system of medical services (conventional medicine and KM). Nonetheless, research on the trend of KM service utilization is scarce.

Against this backdrop, the present study used claims data from the Health Insurance and Review Assessment Service (HIRA) of Korea for the 10 years from 2010 to 2019 to examine the current state and changes in utilization of surgical and non-surgical services for patients with RCST (M751, S460), IS (M754), and AC (M750). Korea has a dual medical system (KM and conventional medicine), and most of the population is enrolled in the National Health Insurance service (NHIS). Therefore, the above data are highly representative and provide information on the use of oriental KM. Accordingly, the objective of this study was to show trends in various treatment modalities, including KM, for shoulder disorders, as well as providing useful basic information and insights for clinicians and policymakers.

## 2. Materials and Methods

### 2.1. Data Source

This study used 10-year (January 2010 to December 2019) claims data from the national patient sample data of HIRA (HIRA-NPS). The health security system of Korea is characterized by the health insurance coverage of the NHIS, which is a mandatory social health insurance with coverage for about 97% of the national population. All healthcare providers (hospitals and clinics) in Korea submit their medical records, including diagnosis and procedure codes, to HIRA to be entitled to reimbursement for the healthcare service they provided. HIRA-NPS data constitute a 2% sample (approximately 1,000,000 people) from sex- and age-stratified random sampling each year, out of the entire Korean population (those enrolled in the NHIS program or Medical Aid). The claims data ensure the representation of the national population and include data for a variety of parameters instrumental for health care research, such as the status of medical services, along with data on diagnosis, treatment/procedure, surgical history, and medication prescription. Thus, claims data are useful in health care or public health research. During sampling, we de-identified the raw data by masking any information that could allow the identification of individuals or entities/corporations. We then statistically processed stratified random samples of one-year segments to obtain secondary data to be used in the analyses of our study.

### 2.2. Study Design and Population

This retrospective, cross-sectional, descriptive study used HIRA-NPS data from January 2010 to December 2019. The study population included patients with AC, IS, and RCST. To identify the study population, we used the diagnosis codes of AC (M750), IS (M754), and RCST (M751, S460) from the seventh revision of the Korean Standard Classification of Diseases (KCD-7). KCD-7 is the Korean version of the International Classification of Disease and Cause of Death-10 (ICD-10). We excluded claims data with information missing on the medical department or health care institution. Only those with the claim submission codes of medical institutions, KM institutions, or community health centers were included.

### 2.3. Study Outcomes

We classified the general demographic characteristics of the study population into the categories of age, sex, type of visit, and type of medical institution. Age was divided into seven 10-year groups from under 20 years old to 70 years old and older. Visit type was classified as outpatient or inpatient. The type of medical institution was classified as hospitals, clinics, KM hospitals, and KM clinics. We analyzed the number of patients, total medical costs, number of claims by category, and medical costs by category for each year from 2010 to 2019.

The total medical costs included costs from total claims incurred during outpatient and inpatient services, and based on the claims issued, they were categorized considering the treatment details: consultation, hospitalization, medication, anesthesia, physical therapy, injections, procedure/surgery, examination, radiological evaluation, and others. Furthermore, we analyzed non-surgical services according to the percentage of prescription of physical therapy, injection therapy, nerve blocks, other non-surgical procedures, and KM therapy, as well as information on the prescription status of medications and surgical treatment. In addition, to examine the monthly trends in healthcare utilization for shoulder disorders, we analyzed the monthly statistics for medical service use for AC, IS, and RCST.

### 2.4. Statistical Analysis

We first performed descriptive statistical analysis. We categorized shoulder disorders into AC, IS, or RCST and expressed the ratio of the number of patients for each type to the total number of patients as percentages (%). For each year, the total numbers of patients with AC, IS, and RCST as a primary diagnosis were expressed as percentages (%). In addition, to examine the monthly trends of medical service utilization for each disorder, we plotted the number of medical service uses for each shoulder disorder, and we presented the average cost per claim by obtaining the total number of claims and costs first, then dividing the costs by the number of claims. Costs were calculated as the total cost of the patient’s out-of-pocket costs and the insurers’ costs.

For each category of non-surgical services, we obtained the number of claims and ratio (%), cost per claim, and status of medical service utilization per shoulder disease type. Likewise, for medications, we obtained the number of claims and ratio (%) (both the total values and the values for each shoulder disease) and cost per claim, as well as annual changes in the patients taking opioids, non-steroidal anti-inflammatory drugs (NSAIDs), muscle relaxants, and systemic steroids. The same information (total and disease-specific number of claims, ratio (%), cost per claim) was obtained for surgical treatment. The category of claim was classified and calculated exactly as it was written in the claim, and the treatment details were reclassified by the researcher by comprehensively considering the procedure code and category written in the claim.

All costs in KRW were converted to USD according to the KRW to USD base exchange rate for each year. The reported values were adjusted according to the 2019 consumer price index in the healthcare service sector. The base exchange rate applied for each year and the consumer price index in the healthcare service sector with reference to 2019 are summarized in Supplementary Table S1. All statistical analyses were performed using SAS 9.4 (SAS Institute Inc., Cary, NC, USA).

## 3. Results

**Figure 1:**
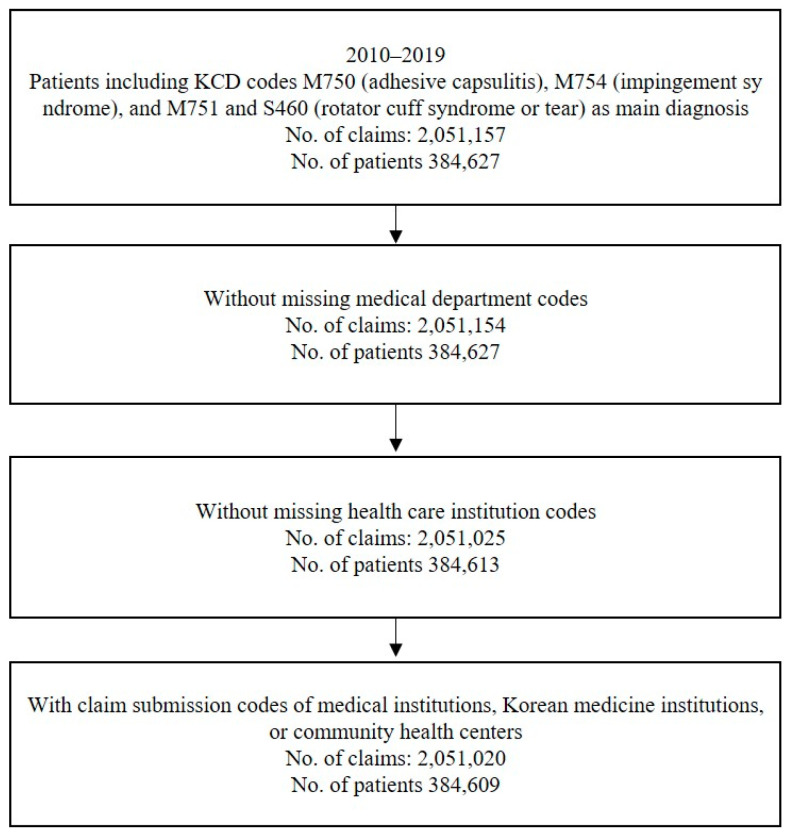
Flow chart of the inclusion of patient data.

The number of patients including KCD codes M750 (adhesive capsulitis), M754 (impingement syndrome), and M751 and S460 (rotator cuff syndrome or tear) as the main diagnosis was 384,627. After excluding several missing values, 384,609 patients and 2,051,020 claims were finally analyzed (Figure 1).

### 3.1. Trend of Health Care Utilization for Shoulder Disorders

**Figure 2:**
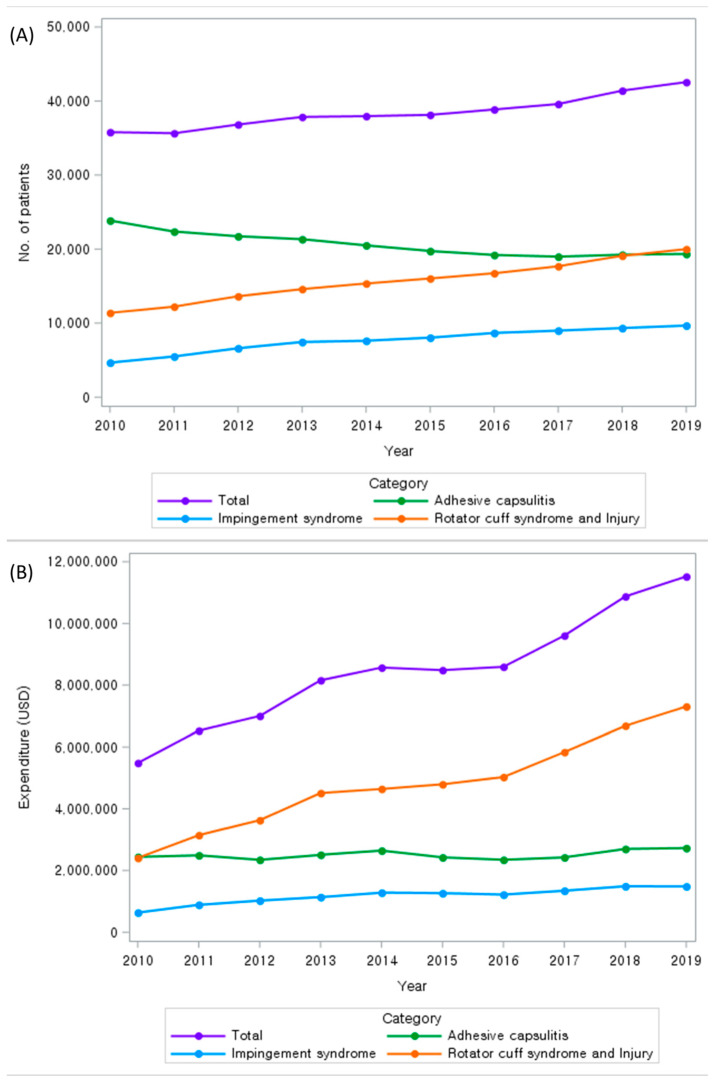
Ten-year (2010–2019) trend of health care utilization for the treatment of common shoulder disorders. (**A**) Number of patients in total and by shoulder disorder type, (**B**) medical costs in total and by shoulder disorder type.

The 10-year trend of healthcare utilization for patients with shoulder disorders in Korea is illustrated in Figure 2, which shows a steady increase in the total number of patients and medical costs for common shoulder disorders. Notably, the number of patients with AC decreased over the 10 years. In addition, the increment in medical costs was larger than the increment in number of patients, with the total medical costs more than doubling over the 10 years. In particular, the medical costs of patients with RCST more than tripled. More details on yearly data on the total number of patients and medical costs can be found in Supplementary Table S1.

### 3.2. General Characteristics of the Study Population

**Figure 3:**
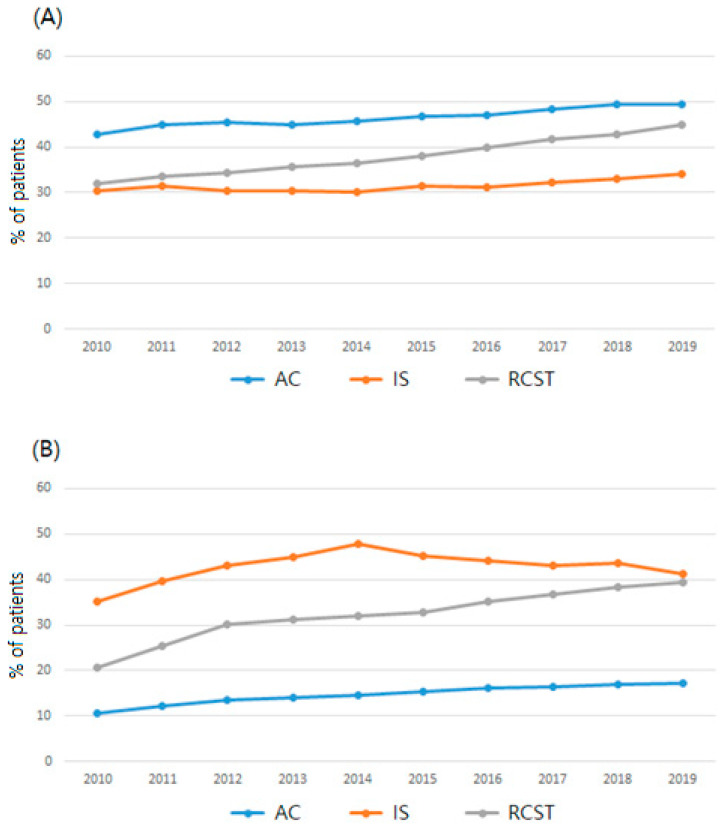
Ten-year (2010–2019) trend of health care utilization by type of shoulder disorder. (**A**) Percentage of patients aged 60 years or older by shoulder disorder type by year, (**B**) percentage of patients visiting hospitals (hospital-level medical institutions) by shoulder disorder type by year. Note: AC, adhesive capsulitis; IS, impingement syndrome; RCST, rotator cuff syndrome or tear.

Table 1 presents the general characteristics of patients who visited medical institutions with AC, IS, or RCST as their primary diagnosis for the period 2010–2019. Trends regarding the general characteristics of patients with shoulder disorders from 2010 to 2019 are shown in Figure 3 and Supplementary Tables S2–S4.

### 3.3. Ten-Year Trend of Monthly Claims

The 10-year trend of monthly claims from 2010 to 2019 is shown in Supplementary Figure S1. Examining the status by month, healthcare utilization for shoulder disorders was the most frequent in March. AC showed the largest number of claims in March for all years in the study period apart from 2013 and 2015 (Figure S1A), while for IS and RCST, it was all years in the study period apart from 2013 (Figure S1B,C).

### 3.4. Claims and Medical Costs by Claims Category

For each type of shoulder disorder, the details of the claims and costs per category of claims are presented in Table 2. Consultation was the category accounting for the highest number of claims for all patients with AC, IS, and RCST. For AC, IS, and RCST, the frequently used services were injections and physical therapy. As for medical costs, in the case of AC and IS, the cost of consultation was the highest, showing the same trend as the number of claims, whereas in the case of RCST, the cost of hospitalization was the highest, showing a discrepancy from the results on the number of claims.

### 3.5. Treatment Details: Non-Surgical Services

Table 3 outlines the information on the number of claims and the average cost per claim for detailed categories of non-surgical treatment. Physical therapy was the most frequently used non-surgical service accounted for. As for detailed usage of injection therapy, subcutaneous or intramuscular injection was used the most, followed by intra-articular injection. Among types of nerve blocks, suprascapular nerve block and axillary block were frequently administered. As for the detailed usage of KM therapy, acupuncture was the most frequently used service, and its cost per claim was lower than that of other items of treatment. The yearly trends for detailed usage of non-surgical services are presented in Supplementary Table S5. The usage of nerve blocks more than doubled from 2010 to 2019, whereas the utilization of KM therapy showed a decreasing trend over 10 years.

### 3.6. Treatment Details: Medications

**Figure 4:**
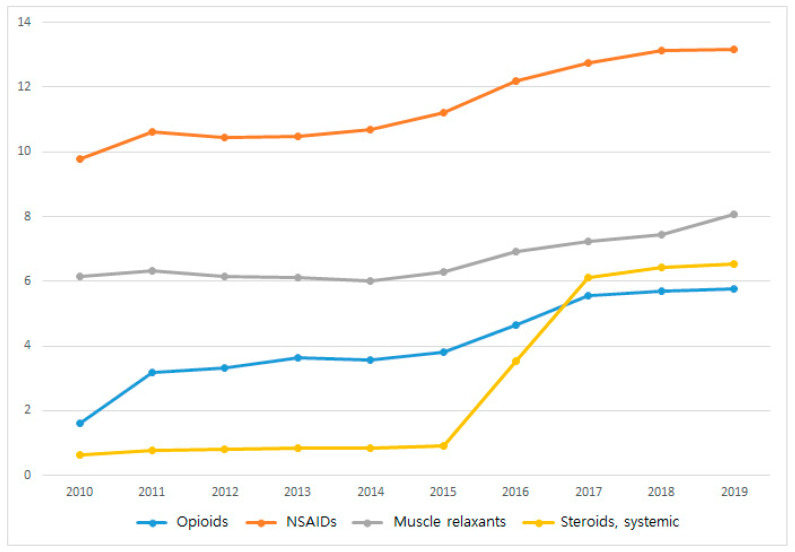
Ten-year (2010–2019) trend of medication use from claims data: opioids, NSAIDs, muscle relaxants, and systemic steroids.

Table 4 shows the total and disease-specific percentages of claims for opioids, non-opioid pain relief medication, anesthetic agents, gastrointestinal agents, antipsychotics, antibiotics, and steroids. The prescription of opioids showed no disease-specific differences. Figure 4 shows the annual trend in the prescription of opioids, NSAIDs, muscle relaxants, and systemic steroids. Prescription rates for all types of medications showed an increasing trend for the 10 years. In particular, the prescription of opioid analgesics more than tripled. Information on the yearly figures of medication claims and their percentages are presented in Supplementary Table S6.

### 3.7. Treatment Details: Surgical Services

Table 5 gives the results on the number of claims, percentages, and average cost per claim. The percentage of claims prescribed with surgery indicated that the percentage of surgical treatment was the highest for rotator cuff disorders. Among all the claims of surgical treatment, acromioplasty and repair of ruptured shoulder rotator cuff accounted for 92.6%. Regarding the yearly usage of surgical treatment, apart from 2016, the number of claims for acromioplasty and repair of ruptured shoulder rotator cuff increased. For RCST only, the number of claims for these two types of surgery showed a steadily increasing trend during the study period, excluding 2014. Information on the yearly figures of surgical treatment prescription and their percentages are presented in Supplementary Table S7.

## 4. Discussion

This study analyzed the trend of healthcare utilization for patients diagnosed with common shoulder disorders (AC [M750], IS [M754], and RCST [M751, S460]) based on HIRA claims data (HIRA-NPS) from 2010 to 2019 to show trends in various treatment modalities, including KM.

### 4.1. Main Results and Overall Trends

The overall trend showed that the number of patients with shoulder disorders and the associated medical costs were increasing. Notably, while the number of patients with AC, also known as frozen shoulder, decreased, the number of patients with IS and rotator cuff syndrome increased. As for the trends in medical costs, both IS and AC showed no significant changes for the 10-year period, whereas the costs associated with rotator cuff disorders more than tripled. The number of patients with rotator cuff disorders and the claims for surgery more than doubled in Korea. A similar trend was reported in the US: among commercially insured patients aged from 18 to 64 years, the percentage of rotator cuff-related surgery increased by 1.6% per year from 2007 to 2016, and the cost per surgery also increased [26].

Most of the patients were aged 40 years or older for all three types of shoulder disorders. The 50–59 years age group accounted for the highest proportion. Thus, middle-aged or older individuals are frequently affected by shoulder disorders. In particular, the proportion of patients aged 60 years or older showed a steady increase over the 10-year study period. This trend may be attributable to the instability in the anatomical structures of shoulder joints, which makes them prone to degeneration following overuse of the joint, and an increase in the middle-aged or older population owing to the aging of society [9,27]. A study conducted in Korea on the prevalence of shoulder osteoarthritis among older adults reported that aging is a risk factor not only for shoulder osteoarthritis but also for arthritis in other joints [28]. As for sex-related trends, three shoulder disorders had more female patients, and in particular, the proportion of female patients was slightly higher in AC. Women generally show higher healthcare utilization than men owing to their relatively low socioeconomic status and subjective health status [29,30,31]. Differences in the anatomical structure of the shoulder between men and women [32] may contribute to the high healthcare utilization of female patients.

Analyzing the healthcare utilization trend by type of medical institution, patients with IS or rotator cuff-related disorders had higher percentages of using medical services in hospitals compared with patients with AC. An explanation is that the former require detailed imaging evaluations available at hospitals, such as computerized tomography or magnetic resonance imaging. The utilization of medical services at the hospital level also increased over the 10-year study period, which may be associated with an increase in the number of rotator cuff surgeries. Furthermore, an improvement in accessibility of hospitals from the development of high-speed transportation in Korea and increased utilization of hospital-level institutions from the mass preference for general or tertiary hospitals [33] may explain the high utilization rate of hospital-level medical services.

Regarding the monthly trends in medical service usage, claims of healthcare utilization peaked in March. In Korea, the number of people enjoying various types of sports activities has increased since the 2000s, triggered by increased incomes and the increasing popularity of leisure activities following the change to the five-day work week from the previous six-day work week [34,35]. Regarding seasonal trends in shoulder and musculoskeletal disorders, a previous study reported that baseball pitchers living in warm-weather climates are more prone to shoulder disorders owing to increased time of play and outdoor exercise, as well as larger shoulder range of motion, compared with those in cold-weather climates [36]. Moreover, pain from musculoskeletal disorders tends to be more severe in winter [37]. Considering these previous findings, the reason for health care utilization peaking in March may be inferred from the fact that those who enjoy outdoor activities or sports, such as tennis, badminton, table tennis, and golf, tend to start going out for these activities from March, when the weather starts to grow warmer. With such an increase in the number of people resuming exercise after winter break, the number of patients with shoulder disorders also increases [38].

### 4.2. Main Results and Treatment Modalities

We found that among the non-surgical treatment modalities, physical therapy accounted for about half of the claims. Specifically, heat/cold therapy and electrotherapy were the most frequently used services. The usage of suprascapular nerve blocks and axillary blocks also showed a continuously increasing trend for the 10 years included in our study. As for the utilization of KM therapy, about 28% of the patients with AC used the services; the utilization rates were insignificant for IS and RCST. KM therapy, particularly acupuncture, has been recognized in international guidelines across the United States, Canada, Australia, and the United Kingdom, particularly for shoulder pain related to RCST and IS [39]. Research indicates that KM therapy, when combined with conventional methods like nerve blocks, significantly benefits patients with chronic shoulder conditions such as AC [40]. Similar improvements have been noted for IS patients undergoing KM therapy [41]. However, while these findings are encouraging, the current body of evidence is still emerging, and certain studies have provided ambiguous outcomes [42]. Therefore, additional research is essential to more thoroughly ascertain the effectiveness of KM therapy for these conditions.

In terms of medications, we observed a noticeable trend of an increase in the prescription rate of opioids and systemic steroids. Although opioids are effective in pain management, they have the risk of side effects [43]. Indeed, studies are underway to ascertain whether the clinical benefits of opioids are worth the risk compared with NSAIDs or other pain relief medications [44]. Opioids are typically associated with side effects such as nausea and vomiting [45]. As for systemic steroids, although effective in reducing generalized inflammation, they have a risk of side effects on the skin and connective tissues [46]. In the Treatment Guidelines for Shoulder Injuries [47] presented by the Department of Labor and Industries of Washington State, NSAIDs or acetaminophen are mainly recommended for prescription as pain relief, whereas systemic steroids are not included in the guidelines. Therefore, a cautious approach is necessary when prescribing medications for shoulder disorders.

Meanwhile, the percentage of claims for surgical treatment showed a steadily increasing trend. Acromioplasty and repair of ruptured shoulder rotator cuff were the two most commonly applied surgical treatments. In patients with RCST, the number of claims increased by about 1000, from 554 surgeries in 2010 to 1409 in 2019. Thus, most of the increase in the surgical treatment of shoulder disorders was for patients with RCST. However, the surgery rate itself for patients with rotator cuff disorders did not increase significantly. This is thought to be due to an increase in the number of patients with rotator cuff-related disorders, who have relatively more indications for surgery, rather than an increase in the surgery rate for shoulder disorders in general. Another point is that the advantages of rotator cuff surgery have increased over time owing to the development of arthroscopic surgery and repair techniques [48]. However, since the percentage of retears after rotator cuff surgery is as high as 11% to 94% [49], decisions on surgical treatment should be made with a cautious approach.

### 4.3. Strengths and Limitations

This study had a number of limitations. First, given our use of secondary data (claims data for national health insurance), the analyses had to be limited to the details of health care utilization that could be collected from the claims data. In particular, information on the clinical symptoms and severity of the shoulder disorders could not be collected from the available data. Follow-up research is needed for the analysis of trends in health care utilization, taking into account clinical symptoms and disease severity. Second, our analysis could only account for shoulder disorders claimed as a primary diagnosis. Thus, even if the patient received treatment for other comorbidities, analysis on these cases could not be performed, and treatment information for other diseases (i.e., not shoulder diseases) may have been included in the statistics. Third, since analyses were performed only based on data for benefit services, the status of non-benefit services could not be analyzed, which may have led to an underestimation of the costs for individual patients. Lastly, the data used in this study were cross-sectional data in one-year segments, which allowed analysis of overall status and trends but limited analysis of individual changes or long-term trends.

Nonetheless, our study is significant because it performed an analysis of recent trends in the healthcare utilization of patients with AC, IS, and RCST for ten years (2010–2019) and presented various treatment modalities, including physical therapy, injection, surgery, and KM therapy, etc. It is different from previous studies because the analyzed data are highly representative and contain diverse and detailed treatment details. According to recent trends, the number of patients with shoulder disorders and the resulting medical costs may increase in the future. Therefore, it will be important to analyze the cost-effectiveness of the various treatment methods covered in this paper, reflect them in insurance, and ensure that patients receive appropriate treatment.

## 5. Conclusions

This study analyzed the ten-year trend (2010–2019) of healthcare utilization in patients with shoulder disorders, namely AC, IS, and RCST, in Korea based on HIRA-NPS data. We confirmed increasing trends in terms of both the number of patients with shoulder disorders and medical costs associated with treatment. In particular, the use of nerve blocks and prescription of opioid analgesics increased. The study findings are expected to serve as basic data for clinicians, researchers, and policy makers.

## Figures and Tables

**Table 1:** Basic characteristics of the study population.

	Adhesive Capsulitis (M750)		Impingement Syndrome (M754)		Rotator Cuff Syndrome or Tear (M751, S460)	
	No. of Patients (%)		No. of Patients (%)		No. of Patients (%)	
**Total**	206,307 (100.0)		76,548 (100.0)		156,726 (100.0)	
**Age (years)**						
Under 20	972 (0.5)		734 (1.0)		1695 (1.1)	
20–29	3585 (1.7)		2930 (3.8)		5858 (3.7)	
30–39	9233 (4.5)		7146 (9.2)		11,941 (7.6)	
40–49	34,338 (16.6)		17,260 (22.6)		29,745 (19.0)	
50–59	62,679 (30.4)		24,252 (31.7)		46,959 (30.0)	
60–69	50,199 (24.3)		15,119 (19.8)		35,372 (22.6)	
70 or older	45,301 (22.0)		9107 (11.9)		25,156 (16.0)	
**Sex**						
Male	80,201 (38.9)		35,696 (46.6)		70,296 (44.9)	
Female	126,106 (61.1)		40,852 (53.4)		86,430 (55.1)	
**Type of visit**						
Outpatient	202,964 (98.4)		72,705 (95.0)		142,866 (91.2)	
Inpatients	3343 (1.6)		3843 (5.0)		13,860 (8.8)	
**Medical institution**						
Hospital	30,019 (14.6)		33,042 (43.2)		51,842 (33.1)	
Clinic	130,901 (63.5)		43,265 (56.5)		94,688 (60.4)	
Korean medicine hospital	997 (0.5)		188 (0.3)		1234 (0.8)	
Korean medicine clinic	57,195 (27.7)		1848 (2.4)		18,548 (11.8)	

No., number.

**Table 2:** Number of claims and costs for patients with shoulder disorders by category of claim.

	Adhesive Capsulitis			Impingement Syndrome			Rotator Cuff Syndrome or Tear		
	Claims	Cost		Claims	Cost		Claims	Cost	
	No. of Claims (%)	Total Cost (%)	Cost per Claim	No. of Claims (%)	Total Cost (%)	Cost per Claim	No. of Claims (%)	Total Cost (%)	Cost per Claim
Consultation	1,033,439 (43.5)	9,562,665 (38.2)	9.3	279,123 (42.0)	2,882,521 (26.5)	10.3	727,745 (42.2)	7,423,728 (19.2)	10.2
Hospitalization	3682 (0.2)	831,302 (3.3)	225.8	4313 (0.7)	1,419,147 (13.0)	329.0	17,231 (1.0)	7,828,003 (20.3)	454.3
Medication	77,414 (3.3)	225,343 (0.9)	2.9	12,816 (1.9)	199,372 (1.8)	15.6	43,619 (2.5)	807,210 (2.1)	18.5
Anesthesia	134,560 (5.7)	3,773,040 (15.1)	28.0	49,250 (7.4)	1,654,166 (15.2)	33.6	124,964 (7.3)	5,352,246 (13.9)	42.8
Physical therapy	440,377 (18.5)	2,704,602 (10.8)	6.1	143,423 (21.6)	988,485 (9.1)	6.9	348,907 (20.2)	2,759,509 (7.1)	7.9
Injections	564,241 (23.7)	5,440,426 (21.7)	9.6	94,153 (14.2)	1,109,093 (10.2)	11.8	272,559 (15.8)	4,290,533 (11.1)	15.7
Procedures/Surgery	13,989 (0.6)	507,263 (2.0)	36.3	8281 (1.2)	821,855 (7.6)	99.3	29,830 (1.7)	4,757,077 (12.3)	159.5
Examination	21,245 (0.9)	507,338 (2.0)	23.9	11,932 (1.8)	613,802 (5.64)	51.4	35,847 (2.1)	2,545,672 (6.6)	71.0
Diagnostic radiology	73,599 (3.1)	1,296,301 (5.2)	17.6	52,881 (8.0)	1,000,770 (9.2)	18.9	104,307 (6.1)	2,106,542 (5.5)	20.2
Others	13,739 (0.6)	190,869 (0.8)	13.89	9126 (1.4)	201,361 (1.9)	22.1	19,414 (1.1)	780,411 (2.0)	40.2

No., number.

**Table 3:** Number of claims and average cost per claim by type of non-surgical treatment.

	AC + IS + RCST			AC			IS			RCST	
	No. of Claims (%)		Average Cost per Claim	No. of Claims (%)		Average Cost per Claim	No. of Claims (%)		Average Cost per Claim	No. of Claims (%)	Average Cost per Claim
**Physical therapy**											
Total	949,756 (46.3)		6.9	447,986 (43.1)		6.2	146,599 (52.4)		6.9	355,171 (48.6)	7.9
Heat/cold therapy	886,957 (93.4)		1.5	424,514 (94.8)		1.4	136,830 (93.3)		1.5	325,613 (91.7)	1.6
Electric therapy	758,519 (79.9)		3.7	372,063 (83.1)		3.4	118,407 (80.8)		3.7	268,049 (75.5)	4.0
Trigger point injection therapy	16,460 (1.7)		7.6	7048 (1.6)		7.6	3031 (2.1)		7.3	6381 (1.8)	7.8
Exercise therapy	220,881 (23.3)		6.8	101,639 (22.7)		5.2	26,849 (18.3)		7.5	92,393 (26.0)	8.4
Traction therapy	16,894 (1.8)		7.1	6672 (1.5)		6.4	3552 (2.4)		7.1	6670 (1.9)	7.9
Paraffin bath	7392 (0.8)		2.8	3311 (0.7)		2.7	1156 (0.8)		2.8	2925 (0.8)	3.0
Laser therapy	106,104 (11.2)		5.9	39,815 (8.9)		5.4	17,993 (12.3)		5.9	48,296 (13.6)	6.4
Others	2976 (0.3)		22.9	2332 (0.5)		20.3	156 (0.1)		32.1	488 (0.1)	32.2
**Injection**											
Total	391,414 (19.1)		7.4	203,940 (19.6)		5.7	58,828 (21.0)		8.4	128,646 (17.6)	9.8
SC or IM injection	246,990 (63.1)		1.7	133,805 (65.6)		1.3	32,319 (54.9)		1.7	80,866 (62.9)	2.3
IV injection	29,588 (7.6)		22.0	6785 (3.3)		8.7	5065 (8.6)		20.5	17,738 (13.8)	27.5
Intra-articular injection	115,016 (29.4)		14.6	58,476 (28.7)		14.6	21,515 (36.6)		14.2	35,025 (27.2)	14.7
Perineural injection	12 (0.0)		9.5	10 (0.0)		9.6	1 (0.0)		9.6	1 (0.0)	8.7
Others	29,840 (7.6)		5.8	13,723 (6.7)		5.7	4674 (7.9)		5.7	11,443 (8.9)	6.1
**Nerve block**											
Total	286,656 (14.0)		28.6	127,000 (12.2)		28.1	44,762 (16.0)		29.4	114,894 (15.7)	28.8
Peripheral branch block, scapular nerve	250,210 (87.3)		23.7	113,544 (89.4)		23.4	38,992 (87.1)		23.6	97,674 (85.0)	23.9
Peripheral branch block, axillary nerve	66,196 (23.1)		15.6	28,284 (22.3)		15.4	11,059 (24.7)		15.3	26,853 (23.4)	15.8
Peripheral branch block, others	13,859 (4.8)		19.2	6188 (4.9)		19.3	1991 (4.4)		18.1	5680 (4.9)	19.3
Spinal nerve plexus, root or ganglion block	10,005 (3.5)		67.3	3830 (3.0)		66.7	1972 (4.4)		67.3	4203 (3.7)	68.0
Epidural block	6059 (2.1)		39.6	2070 (1.6)		42.4	1053 (2.4)		43.2	2936 (2.6)	36.3
Others	5249 (1.8)		13.5	848 (0.7)		13.2	797 (1.8)		13.6	3604 (3.1)	13.5
**Other non-surgical procedure**											
Total	1660 (0.1)		101.6	1264 (0.1)		105.6	155 (0.1)		95.6	241 (0.0)	84.4
Brisement force	1576 (94.9)		104.1	1243 (98.3)		106.5	149 (96.1)		97.2	184 (76.3)	93.3
Closed reduction in dislocation, shoulder	24 (1.4)		77.4	3 (0.2)		61.8	2 (1.3)		66.2	19 (7.9)	81.0
Closed reduction and immobilization	62 (3.7)		44.6	19 (1.5)		49.2	5 (3.2)		40.4	38 (15.8)	42.8
**Korean medical therapy**											
Total	389,542 (19.0)		11.8	294,913 (28.4)		11.5	9079 (3.2)		14.3	85,550 (11.7)	12.7
Acupuncture, general	381,385 (97.9)		4.1	288,443 (97.8)		4.0	8866 (97.7)		4.5	84,076 (98.3)	4.3
Acupuncture, special											
Intra-articular	193,010 (49.5)		3.1	153,530 (52.1)		3.1	4046 (44.6)		3.3	35,434 (41.4)	3.1
Intraperitoneum	3214 (0.8)		2.8	2490 (0.8)		2.8	80 (0.9)		3.4	644 (0.8)	2.9
Intervertebral	24,201 (6.2)		3.1	16,962 (5.8)		3.0	641 (7.1)		3.4	6598 (7.7)	3.2
Piercing, multidirectional	143,658 (36.9)		4.6	100,573 (34.1)		4.5	3805 (41.9)		5.3	39,280 (45.9)	4.8
Intraorbital	104 (0.0)		2.8	99 (0.0)		2.8	1 (0.0)		5.7	4 (0.0)	2.8
Intranasal	258 (0.1)		2.6	226 (0.1)		2.6	11 (0.1)		2.9	21 (0.0)	3.0
Others	1792 (0.5)		3.7	1447 (0.5)		3.6	16 (0.2)		4.9	329 (0.4)	4.2
Electroacupuncture	82,425 (21.2)		4.1	58,152 (19.7)		3.9	3110 (34.3)		4.6	21,163 (24.7)	4.4
Moxibustion											
Direct	9427 (2.4)		6.3	7997 (2.7)		6.2	156 (1.7)		5.9	1274 (1.5)	7.1
Indirect	61,776 (15.9)		2.7	47,507 (16.1)		2.6	1936 (21.3)		3.1	12,333 (14.4)	3.1
Cupping therapy											
Dry	97,860 (25.1)		3.8	73,182 (24.8)		3.7	2623 (28.9)		4.5	22,055 (25.8)	4.2
Wet	97,620 (25.1)		6.1	75,057 (25.5)		6.0	2266 (25.0)		7.4	20,297 (23.7)	6.4
Warm/cold therapy	186,581 (47.9)		0.9	141,782 (48.1)		0.9	4380 (48.2)		1.0	40,419 (47.2)	1.0

The percentage (%) in the “Total” section represents the percentage against the total claims; the percentage (%) in each category of treatment detail represents the percentage against the total claims of the applicable category. AC, adhesive capsulitis; IS, impingement syndrome; RCST, rotator cuff syndrome or tear; No., number; SC, subcutaneous; IM, intermuscular: IV, intravenous.

**Table 4:** Number of claims and average cost per claim by type of medication.

	AC + IS + RCST		AC		IS		RCST	
	No. of Claims (%)	Average Cost per Claim	No. of Claims (%)	Average Cost per Claim	No. of Claims (%)	Average Cost per Claim	No. of Claims (%)	Average Cost per Claim
Total	1,006,806 (49.1)	11.9	460,115 (44.3)	8.1	172,319 (61.6)	12.8	374,372 (51.2)	16.3
Opioids	211,499 (21.0)	3.3	90,626 (19.7)	2.3	37,544 (21.8)	3.4	83,329 (22.3)	4.3
Non-opioid pain relief medication								
NSAIDs	656,849 (65.2)	4.3	279,379 (60.7)	3.2	123,702 (71.8)	4.8	253,768 (67.8)	5.3
Others	42,373 (4.2)	2.5	18,140 (3.9)	2.1	6559 (3.8)	2.7	17,674 (4.7)	2.8
Neuralgia medication	1311 (0.1)	3.0	586 (0.1)	2.7	102 (0.1)	2.0	623 (0.2)	3.5
Muscle relaxants	332,289 (33.0)	2.0	149,935 (32.6)	1.6	60,259 (35.0)	2.1	122,095 (32.6)	2.3
Anesthetic	136,134 (13.5)	1.7	50,634 (11.0)	0.5	24,681 (14.3)	1.2	60,819 (16.2)	2.9
Gastrointestinal	640,304 (63.6)	3.6	275,266 (59.8)	2.6	119,682 (69.5)	4.0	245,356 (65.5)	4.6
Antipsychotic	66,707 (6.6)	2.8	34,816 (7.6)	2.7	7258 (4.2)	3.3	24,633 (6.6)	2.8
Antibiotics								
Topical	789 (0.1)	5.5	351 (0.1)	6.1	64 (0.0)	5.4	374 (0.1)	4.8
Systemic	33,181 (3.3)	34.1	11,328 (2.5)	10.8	4510 (2.6)	36.8	17,343 (4.6)	48.7
Steroids								
Topical	1177 (0.1)	2.5	497 (0.1)	2.2	115 (0.1)	3.3	565 (0.2)	2.6
Systemic	117,055 (11.6)	0.5	48,917 (10.6)	0.5	23,334 (13.5)	0.5	44,804 (12.0)	0.5
Others	680,996 (67.6)	5.6	327,284 (71.1)	4.1	113,275 (65.7)	5.5	240,437 (64.2)	7.7

The percentage (%) in the “Total” section represents the percentage against the total claims; the percentage (%) in each category of treatment detail represents the percentage against the total claims of the applicable category. AC, adhesive capsulitis; IS, impingement syndrome; RCST, rotator cuff syndrome or tear; No., number; NSAIDS, nonsteroidal anti-inflammatory drugs.

**Table 5:** Number of claims and average cost per claim by type of meditation.

	AC + IS + RCST		AC		IS		RCTS	
	No. of Claims (%)	Average Cost per Claim	No. of Claims (%)	Average Cost per Claim	No. of Claims (%)	Average Cost per Claim	No. of Claims (%)	Average Cost per Claim
Total	13,635 (0.7)	364.1	909 (0.1)	262.9	2153 (0.8)	313.8	10,573 (1.4)	383.1
Acromioplasty and Repair of Ruptured Shoulder Rotator Cuff	12,625 (92.6)	354.1	597 (65.7)	287.4	2016 (93.6)	301.9	10,012 (94.7)	368.6
Reconstruction of Tendon and Ligament	768 (5.6)	120.7	42 (4.6)	122.3	95 (4.4)	136.5	631 (6.0)	118.2
Excision of Joint, Shoulder	665 (4.9)	223.4	183 (20.1)	247.7	134 (6.2)	222.2	348 (3.3)	211.0
Excision of Joint, Others	39 (0.3)	230.0	4 (0.4)	229.4	7 (0.3)	259.4	28 (0.3)	222.7
Replacement Arthroplasty, Shoulder	218 (1.6)	594.5	1 (0.1)	528.2	2 (0.1)	550.8	215 (2.0)	595.2
Removal of Implant for Internal Fixation	60 (0.4)	132.1	7 (0.8)	207.8	11 (0.5)	99.5	42 (0.4)	128.0
Open Reduction of Dislocation	2 (0.0)	326.6	-	-	-	-	2 (0.0)	326.6
Others	732 (5.4)	144.9	142 (15.6)	98.7	146 (6.8)	138.7	444 (4.2)	161.8

The percentage (%) in the “Total” section represents the percentage against the total claims; the percentage (%) in each category of treatment detail represents the percentage against the total claims of the applicable category. AC, adhesive capsulitis; IS, impingement syndrome; RCST, rotator cuff syndrome or tear; No., number; NSAIDS, nonsteroidal anti-inflammatory drugs.

## Data Availability

HIRA data are third-party data not owned by the authors. Raw data are made available from the Health Insurance Review and Assessment Service (HIRA) in Korea.

## Supplementary Materials

The following supporting information can be downloaded at: https://www.mdpi.com/article/10.3390/medicina60050744/s1, Figure S1: Ten-year (2010–2019) trend on monthly claims of medical services for shoulder disorders by age group. (A) adhesive capsulitis, (B) impingement syndrome, (C) rotator cuff syndrome or tear. Asterisk means March in each year, Table S1: General medical service use for patients with shoulder diseases in Korea, Table S2: General medical service use for Adhesive Capsulitis (M750) patients in Korea, Table S3: General medical service use for Impingement Syndrome (M754) patients in Korea, Table S4: General medical service use for Rotator Cuff Tear or Syndrome (M751, S460) patients in Korea, Table S5: Annual non-surgical treatment prescription rate, Table S6: Annual medication prescription rate, Table S7: Annual surgical treatment prescription rate, Table S8: Annual surgery rate for rotator cuff syndrome or tear (M751, S460).
