# Supplementary material for: Health Care Utilization for Common Shoulder Disorders: Analysis of the 2010–2019 National Patient Sample Data from the Health Insurance Review and Assessment Service in Korea
Source: Medicina (Kaunas). 2024 Apr 29;60(5):744. doi: 10.3390/medicina60050744 (PMC11122813; doi:10.3390/medicina60050744)
Supplement: Supplementary file 1 [file medicina-60-00744-s001.zip › medicina-2968159-supplementary.pdf]

Supplement Table S1. General medical service use for patients with shoulder diseases in Korea.

	2010	2011	2012	2013	2014	2015	2016	2017	2018	2019
**Number of patients**	35,798	35,648	36,823	37,852	37,956	38,120	38,861	39,593	41,400	42,558
AC	23,854	22,369	21,729	21,337	20,507	19,741	19,206	18,978	19,240	19,346
IS	4,666	5,501	6,607	7,456	7,612	8,035	8,675	8,983	9,340	9,673
RCST	11,390	12,220	13,628	14,601	15,355	16,023	16,735	17,683	19,097	19,994
**Total claims**	182,746	183,150	201,408	209,085	209,140	207,823	206,459	210,732	216,100	224,377
AC	122,821	114,141	112,489	109,946	106,921	100,781	95,413	92,232	92,302	92,515
IS	16,089	19,121	25,216	28,585	28,437	29,503	32,060	32,152	33,655	35,093
RCST	43,836	49,888	63,703	70,554	73,782	77,539	78,986	86,348	90,143	96,769
**Total expenditure†**	5,485,196	6,528,318	7,003,404	8,158,615	8,570,579	8,486,077	8,593,962	9,607,591	10,875,282	11,522,543
AC	2,440,371	2,493,999	2,346,401	2,508,789	2,645,057	2,426,660	2,345,948	2,426,024	2,699,556	2,727,445
IS	636,430	888,134	1,025,967	1,138,625	1,282,235	1,266,203	1,218,521	1,343,810	1,491,269	1,486,115
RCST	2,408,395	3,146,184	3,631,036	4,511,201	4,643,288	4,793,214	5,029,494	5,837,757	6,684,457	7,308,983
**Avg. expenditure per patient**	153.2	183.1	190.2	215.5	225.8	222.6	221.2	242.7	262.7	270.8
AC	102.3	111.5	108.0	117.6	129.0	122.9	122.2	127.8	140.3	141.0
IS	136.4	161.5	155.3	152.7	168.5	157.6	140.5	149.6	159.7	153.6
RCST	211.5	257.5	266.4	309.0	302.4	299.2	300.5	330.1	350.0	365.6
**Avg. expenditure per claim†**	30.0	35.6	34.8	39.0	41.0	40.8	41.6	45.6	50.3	51.4
AC	19.9	21.9	20.9	22.8	24.7	24.1	24.6	26.3	29.3	29.5
IS	39.6	46.5	40.7	39.8	45.1	42.9	38.0	41.8	44.3	42.4
RCST	54.9	63.1	57.0	63.9	62.9	61.8	63.7	67.6	74.2	75.5

AC, adhesive capsulitis; IS, impingement syndrome; RCST, rotator cuff syndrome or tear;

Supplement Table S2. General medical service use for Adhesive Capsulitis (M750) patients in Korea.

	Adhesive Capsulitis (M750)									
	**2010**	**2011**	**2012**	**2013**	**2014**	**2015**	**2016**	**2017**	**2018**	**2019**
	**N (%)**	**N (%)**	**N (%)**	**N (%)**	**N (%)**	**N (%)**	**N (%)**	**N (%)**	**N (%)**	**N (%)**
**Total**	23,854(100.0)	22,369(100.0)	21,729(100.0)	21,337(100.0)	20,507(100.0)	19,741(100.0)	19,206(100.0)	18,978(100.0)	19,240(100.0)	19,346(100.0)
**AGE**										
Under 20	177(0.7)	148(0.7)	109(0.5)	111(0.5)	96(0.5)	87(0.4)	73(0.4)	53(0.3)	62(0.3)	56(0.3)
20-29	624(2.6)	468(2.1)	453(2.1)	384(1.8)	366(1.8)	306(1.6)	267(1.4)	266(1.4)	230(1.2)	221(1.1)
30-39	1,495(6.3)	1,237(5.5)	1,084(5.0)	999(4.7)	939(4.6)	793(4.0)	781(4.1)	675(3.6)	669(3.5)	561(2.9)
40-49	4,641(19.5)	4,035(18.0)	3,675(16.9)	3,629(17.0)	3,349(16.3)	3,148(16.0)	3,072(16.0)	2,938(15.5)	2,954(15.4)	2,897(15.0)
50-59	6,717(28.2)	6,455(28.9)	6,524(30.0)	6,653(31.2)	6,405(31.2)	6,196(31.4)	5,971(31.1)	5,874(31.0)	5,837(30.3)	6,047(31.3)
60-69	5,699(23.9)	5,472(24.5)	5,245 (24.1)	4,889(22.9)	4,667(22.8)	4,684(23.7)	4,705(24.5)	4,805(25.3)	5,010(26.0)	5,023(26.0)
70 or older	4,501(18.9)	4,554(20.4)	4,639(21.4)	4,672(21.9)	4,685(22.9)	4,527(22.9)	4,337(22.6)	4,367(23.0)	4,478(23.3)	4,541(23.5)
**Gender**										
Male	8,875(37.2)	8,319(37.2)	8,345(38.4)	8,281(38.8)	7,873(38.4)	7,773(39.4)	7,528(39.2)	7,600(40.1)	7,712(40.1)	7,895(40.8)
Female	14,979(62.8)	14,050(62.8)	13,384(61.6)	13,056(61.2)	12,634(61.6)	11,968(60.6)	11,678(60.8)	11,378(60.0)	11,528(59.9)	11,451(59.2)
**Type of visit**										
outpatients	23,655(99.2)	22,159(99.1)	21,473(98.8)	21,011(98.5)	20,134(98.2)	19,330(97.9)	18,831(98.1)	18,624(98.1)	18,820(97.8)	18,927(97.8)
inpatients	199(0.8)	210(0.9)	256(1.2)	326(1.5)	373(1.8)	411(2.1)	375(2.0)	354(1.9)	420(2.2)	419(2.2)
**Medical institution**										
Hospital	2,509(10.5)	2,704(12.1)	2,948(13.6)	2,996(14.0)	2,964(14.5)	3,035(15.4)	3,126(16.3)	3,136(16.5)	3,261(17.0)	3,340(17.3)
Clinic	12,956(54.3)	13,355(59.7)	13,359(61.5)	13,210(61.9)	12,806(62.5)	12,628(64.0)	12,771(66.5)	13,015(68.6)	13,281(69.0)	13,520(69.9)
KM hospital	166(0.7)	117(0.5)	93(0.4)	102(0.5)	71(0.4)	81(0.4)	118(0.6)	85(0.5)	84(0.4)	80(0.4)
KM clinic	9,939(41.7)	7,687(34.4)	6,763(31.1)	6,419(30.1)	5,956(29.0)	5,208(26.4)	4,295(22.4)	3,787(20.0)	3,722(19.4)	3,419(17.7)

Supplement Table S3. General medical service use for Impingement Syndrome (M754) patients in Korea.

	Impingement Syndrome (M754)									
	2010	2011	2012	2013	2014	2015	2016	2017	2018	2019
	N (%)	N (%)	N (%)	N (%)	N (%)	N (%)	N (%)	N (%)	N (%)	N (%)
**Total**	4,666(100.0)	5,501(100.0)	6,607(100.0)	7,456(100.0)	7,612(100.0)	8,035(100.0)	8,675(100.0)	8,983(100.0)	9,340(100.0)	9,673(100.0)
**AGE**										
Under 20	41(0.9)	43(0.8)	74(1.1)	73(1.0)	80(1.1)	81(1.0)	84(1.0)	83(0.9)	86(0.9)	89(0.9)
20-29	180(3.9)	175(3.2)	210(3.2)	261(3.5)	265(3.5)	290(3.6)	367(4.2)	367(4.1)	402(4.3)	413(4.3)
30-39	483(10.4)	517(9.4)	644(9.8)	724(9.7)	704(9.3)	754(9.4)	797(9.2)	792(8.8)	837(9.0)	894(9.2)
40-49	1,126(24.1)	1,321(24.0)	1,573(23.8)	1,677(22.5)	1,761(23.1)	1,853(23.1)	1,938(22.3)	1,971(21.9)	2,060(22.1)	1,980(20.5)
50-59	1,420(30.4)	1,718(31.2)	2,096(31.7)	2,447(32.8)	2,505(32.9)	2,538(31.6)	2,792(32.2)	2,863(31.9)	2,874(30.8)	2,999(31.0)
60-69	891(19.1)	1,085(19.7)	1,248(18.9)	1,396(18.7)	1,398(18.4)	1,561(19.4)	1,686(19.4)	1,843(20.5)	1,927(20.6)	2,084(21.5)
70 or older	525(11.3)	642(11.7)	762(11.5)	878(11.8)	899(11.8)	958(11.9)	1,011(11.7)	1,064(11.8)	1,154(12.4)	1,214(12.6)
**Gender**										
Male	2,137(45.8)	2,502(45.5)	2,981(45.1)	3,529(47.3)	3,529(46.4)	3,751(46.7)	4,050(46.7)	4,297(47.8)	4,346(46.5)	4,574(47.3)
Female	2,529(54.2)	2,999(54.5)	3,626(54.9)	3,927(52.7)	4,083(53.6)	4,284(53.3)	4,625(53.3)	4,686(52.2)	4,994(53.5)	5,099(52.7)
**Type of visit**										
outpatients	4,515(96.8)	5,254(95.5)	6,267(94.9)	7,065(94.8)	7,126(93.6)	7,531(93.7)	8,216(94.7)	8,561(95.3)	8,886(95.1)	9,284(96.0)
inpatients	151(3.2)	247(4.5)	340(5.2)	391(5.2)	486(6.4)	504(6.3)	459(5.3)	422(4.7)	454(4.9)	389(4.0)
**Medical institution**										
Hospital	1,639(35.1)	2,181(39.7)	2,840(43.0)	3,355(45.0)	3,630(47.7)	3,629(45.2)	3,835(44.2)	3,877(43.2)	4,075(43.6)	3,981(41.2)
Clinic	2,852(61.1)	3,230(58.7)	3,684(55.8)	4,055(54.4)	3,994(52.5)	4,420(55.0)	4,858(56.0)	5,157(57.4)	5,283(56.6)	5,732(59.3)
KM hospital	13(0.3)	14(0.3)	8(0.1)	17(0.2)	14(0.2)	17(0.2)	30(0.4)	16(0.2)	28(0.3)	31(0.3)
KM clinic	238(5.1)	176(3.2)	226(3.4)	203(2.7)	189(2.5)	152(1.9)	183(2.1)	143(1.6)	179(1.9)	159(1.6)

Supplement Table S4. General medical service use for Rotator Cuff Tear or Syndrome (M751, S460) patients in Korea.

	Rotator Cuff Syndrome and Tear (M751, S460)									
	**2010**	**2011**	**2012**	**2013**	**2014**	**2015**	**2016**	**2017**	**2018**	**2019**
	**N (%)**	**N (%)**	**N (%)**	**N (%)**	**N (%)**	**N (%)**	**N (%)**	**N (%)**	**N (%)**	**N (%)**
**Total**	11,390(100.0)	12,220(100.0)	13,628(100.0)	14,601(100.0)	15,355(100.0)	16,023(100.0)	16,735(100.0)	17,683(100.0)	19,097(100.0)	19,994(100.0)
**AGE**										
Under 20	194(1.7)	168(1.4)	173(1.3)	182(1.3)	181(1.2)	178(1.1)	158(0.9)	133(0.8)	174(0.9)	154(0.8)
20-29	605(5.3)	538(4.4)	551(4.0)	602(4.1)	610(4.0)	603(3.8)	593(3.5)	567(3.2)	594(3.1)	595(3.0)
30-39	1,183(10.4)	1,176(9.6)	1,181(8.7)	1,170(8.0)	1,251(8.2)	1,208(7.5)	1,201(7.2)	1,171(6.6)	1,198(6.3)	1,202(6.0)
40-49	2,619(23.0)	2,668(21.8)	2,855(21.0)	2,897(19.8)	3,043(19.8)	3,068(19.2)	3,007(18.0)	3,073(17.4)	3,302(17.3)	3,213(16.1)
50-59	3,148(27.6)	3,585(29.3)	4,176(30.6)	4,546(31.1)	4,653(30.3)	4,885(30.5)	5,091(30.4)	5,374(30.4)	5,659(29.6)	5,842(29.2)
60-69	2,246(19.7)	2,527(20.7)	2,782(20.4)	2,967(20.3)	3,253(21.2)	3,445(21.5)	3,866(23.1)	4,305(24.4)	4,763(24.9)	5,218(26.1)
70 or older	1,395(12.3)	1,558(12.8)	1,910(14.0)	2,237(15.3)	2,364(15.4)	2,636(16.5)	2,819(16.8)	3,060(17.3)	3,407(17.8)	3,770(18.9)
**Gender**										
Male	4,782(42.0)	5,254(43.0)	5,896(43.3)	6,513(44.6)	6,911(45.0)	7,312(45.6)	7,482(44.7)	8,206(46.4)	8,763(45.9)	9,177(45.9)
Female	6,608(58.0)	6,966(57.0)	7,732(56.7)	8,088(55.4)	8,444(55.0)	8,711(54.4)	9,253(55.3)	9,477(53.6)	10,334(54.1)	10,817(54.1)
**Type of visit**										
outpatients	10,739(94.3)	11,343(92.8)	12,554(92.1)	13,295(91.1)	14,002(91.2)	14,484(90.4)	15,115(90.3)	15,956(90.2)	17,313(90.7)	18,065(90.4)
inpatients	651(5.7)	877(7.2)	1,074(7.9)	1,306(8.9)	1,353(8.8)	1,539(9.6)	1,620(9.7)	1,727(9.8)	1,784(9.3)	1,929(9.7)
**Medical institution**										
Hospital	2,356(20.7)	3,106(25.4)	4,093(30.0)	4,560(31.2)	4,910(32.0)	5,264(32.9)	5,889(35.2)	6,483(36.7)	7,297(38.2)	7,884(39.4)
Clinic	6,589(57.9)	7,116(58.2)	7,887(57.9)	8,644(59.2)	9,210(60.0)	9,800(61.2)	10,489(62.7)	11,076(62.6)	11,720(61.4)	12,157(60.8)
KM hospital	18(0.2)	28(0.2)	50(0.4)	48(0.3)	136(0.9)	160(1.0)	180(1.1)	182(1.0)	219(1.2)	213(1.1)
KM clinic	2,859(25.1)	2,521(20.6)	2,348(17.2)	2,200(15.1)	2,006(13.1)	1,769(11.0)	1,265(7.6)	1,132(6.4)	1,220(6.4)	1,228(6.1)

Supplement Table S5. Annual non-surgical treatment prescription rate.

	2010	2011	2012	2013	2014	2015	2016	2017	2018	2019
	**N (%)**	**N (%)**	**N (%)**	**N (%)**	**N (%)**	**N (%)**	**N (%)**	**N (%)**	**N (%)**	**N (%)**
**Physical Therapy**										
Total	75,589(41.4)	80,858(44.1)	94,983(47.2)	99,370(47.5)	99,331(47.5)	97,102(46.7)	96,742(46.9)	100,107(47.5)	101,450(46.9)	104,224(46.5)
heat/cold therapy	73,334(97.0)	77,968(96.4)	89,612(94.3)	92,034(92.6)	93,182(93.8)	91,109(93.8)	90,656(93.7)	88,609(88.5)	94,558(93.2)	95,895(92.0)
electric therapy	63,532(84.0)	67,090(83.0)	77,530(81.6)	81,526(82.0)	80,355(80.9)	76,593(78.9)	75,492(78.0)	77,348(77.3)	78,845(77.7)	80,208(77.0)
TPI therapy	1,305(1.7)	1,468(1.8)	1,571(1.7)	1,644(1.7)	1,592(1.6)	1,681(1.7)	1,695(1.8)	1,824(1.8)	1,742(1.7)	1,938(1.9)
exercise therapy	13,794(18.2)	15,563(19.2)	24,785(26.1)	24,904(25.1)	23,698(23.9)	23,710(24.4)	22,778(23.5)	24,470(24.4)	24,059(23.7)	23,120(22.2)
traction therapy	871(1.2)	897(1.1)	1,154(1.2)	1,792(1.8)	1,516(1.5)	1,643(1.7)	1,769(1.8)	2,145(2.1)	2,480(2.4)	2,627(2.5)
paraffin bath	701(0.9)	579(0.7)	709(0.7)	578(0.6)	757(0.8)	690(0.7)	804(0.8)	868(0.9)	741(0.7)	965(0.9)
laser therapy	6,434(8.5)	6,834(8.5)	9,000(9.5)	9,359(9.4)	9,367(9.4)	10,690(11.0)	11,734(12.1)	12,963(12.9)	13,960(13.8)	15,763(15.1)
others	136(0.2)	167(0.2)	298(0.3)	264(0.3)	327(0.3)	308(0.3)	225(0.2)	230(0.2)	444(0.4)	577(0.6)
**Injection**										
Total	37,740(20.7)	38,424(21.0)	40,959(20.3)	39,769(19.0)	38,839(18.6)	38,648(18.6)	38,087(18.4)	38,554(18.3)	40,064(18.5)	40,330(18.0)
SC or IM Injection	30,170(79.9)	28,888(75.2)	28,351(69.2)	26,238(66.0)	24,748(63.7)	23,190(60.0)	22,480(59.0)	21,464(55.7)	21,715(54.2)	19,746(49.0)
IV Injection	1,577(4.2)	2,014(5.2)	2,406(5.9)	2,787(7.0)	3,151(8.1)	3,400(8.8)	3,341(8.8)	3,462(9.0)	3,677(9.2)	3,773(9.4)
Intraarticular Injection	5,950(15.8)	7,623(19.8)	10,106(24.7)	10,848(27.3)	11,156(28.7)	12,292(31.8)	12,684(33.3)	13,723(35.6)	14,581(36.4)	16,053(39.8)
Perineural Injection	8(0.0)	2(0.0)	-	-	-	1(0.0)	-	1(0.0)	-	-
Others	2,031(5.4)	2,223(5.8)	2,752(6.7)	2,802(7.0)	2,912(7.5)	3,169(8.2)	3,072(8.1)	3,270(8.5)	3,535(8.8)	4,074(10.1)
**Nerve block**										
Total	18,236(10.0)	20,448(11.2)	23,021(11.4)	25,926(12.4)	26,888(12.9)	29,300(14.1)	30,700(14.9)	35,623(16.9)	36,880(17.1)	39,634(17.7)
Peripheral Branch Block, Scapular Nerve	16,346(89.6)	18,409(90.0)	20,585(89.4)	23,056(88.9)	23,999(89.3)	26,074(89.0)	26,878(87.6)	30,576(85.8)	30,998(84.1)	33,289(84.0)
Peripheral Branch Block, Axillary Nerve	2,799(15.3)	3,239(15.8)	4,378(19.0)	5,252(20.3)	5,418(20.2)	6,089(20.8)	7,453(24.3)	9,335(26.2)	10,270(27.8)	11,963(30.2)
Peripheral Branch Block, Others	959(5.3)	998(4.9)	1,090(4.7)	1,205(4.6)	1,245(4.6)	1,378(4.7)	1,502(4.9)	1,664(4.7)	1,794(4.9)	2,024(5.1)
Spinal Nerve Plexus, Root or Ganglion Block	178(1.0)	265(1.3)	288(1.3)	515(2.0)	650(2.4)	934(3.2)	1,072(3.5)	1,498(4.2)	1,941(5.3)	2,664(6.7)
Epidural Block	294(1.6)	332(1.6)	529(2.3)	567(2.2)	641(2.4)	828(2.8)	757(2.5)	601(1.7)	754(2.0)	756(1.9)
Others	80(0.4)	77(0.4)	49(0.2)	93(0.4)	157(0.6)	157(0.5)	137(0.4)	1,308(3.7)	1,554(4.2)	1,637(4.1)

**Other nonsurgical procedure**										
Total	96(0.1)	122(0.1)	121(0.1)	147(0.1)	143(0.1)	163(0.1)	181(0.1)	222(0.1)	218(0.1)	247(0.1)
Brisement Force	82(85.4)	118(96.7)	114(94.2)	134(91.2)	137(95.8)	158(96.9)	170(93.9)	211(95.0)	211(96.8)	241(97.6)
Closed Reduction of Dislocation, Shoulder	4(4.2)	-	1(0.8)	4(2.7)	2(1.4)	1(0.6)	4(2.2)	3(1.4)	2(0.9)	3(1.2)
Closed Reduction and Immobilization	10(10.4)	4(3.3)	6(5.0)	9(6.1)	5(3.5)	4(2.5)	8(4.4)	8(3.6)	5(2.3)	3(1.2)
**Korean Medical Therapy**										
Total	60,997(33.4)	50,593(27.6)	43,449(21.6)	43,229(20.7)	40,685(19.5)	37,588(18.1)	32,059(15.5)	27,675(13.1)	27,658(12.8)	25,609(11.4)
Acupuncture, general	60,526(99.2)	49,525(97.9)	42,751(98.4)	42,107(97.4)	39,533(97.2)	37,014(98.5)	30,702(95.8)	27,051(97.7)	27,009(97.7)	25,167(98.3)
Acupuncture, special										
Intraarticular	33,923(55.6)	29,874(59.0)	24,513(56.4)	20,967(48.5)	18,537(45.6)	18,767(49.9)	13,435(41.9)	11,134(40.2)	11,805(42.7)	10,055(39.3)
Intraperitoneum	265(0.4)	490(1.0)	203(0.5)	244(0.6)	413(1.0)	345(0.9)	341(1.1)	312(1.1)	322(1.2)	279(1.1)
Intervertebral	2,336(3.8)	1,560(3.1)	1,867(4.3)	3,070(7.1)	2,359(5.8)	1,866(5.0)	2,866(8.9)	3,069(11.1)	2,643(9.6)	2,565(10.0)
Piercing, multidirectional	20,808(34.1)	15,917(31.5)	14,112(32.5)	16,057(37.1)	16,468(40.5)	13,539(36.0)	12,263(38.3)	10,692(38.6)	11,828(42.8)	11,974(46.8)
Intraorbital	36(0.1)	1(0.0)	1(0.0)	9(0.0)	36(0.1)	1(0.0)	-	10(0.0)	10(0.0)	-
Intranasal	24(0.0)	29(0.1)	14(0.0)	43(0.1)	21(0.1)	38(0.1)	5(0.0)	26(0.1)	52(0.2)	6(0.0)
others	397(0.7)	432(0.9)	298(0.7)	117(0.3)	94(0.2)	79(0.2)	70(0.2)	75(0.3)	168(0.6)	62(0.2)
Electroacupuncture	8,926(14.6)	8,748(17.3)	7,437(17.1)	8,226(19.0)	8,316(20.4)	8,327(22.2)	7,699(24.0)	7,291(26.3)	9,173(33.2)	8,282(32.3)
Moxibustion										
Direct	959(1.6)	1,117(2.2)	882(2.0)	1,054(2.4)	1,030(2.5)	996(2.6)	920(2.9)	942(3.4)	877(3.2)	650(2.5)
Indirect	9,223(15.1)	8,372(16.5)	7,305(16.8)	6,161(14.3)	5,712(14.0)	4,842(12.9)	5,269(16.4)	4,681(16.9)	5,382(19.5)	4,829(18.9)
Cupping therapy										
Dry	12,300(20.2)	11,023(21.8)	10,785(24.8)	10,854(25.1)	11,260(27.7)	9,023(24.0)	9,611(30.0)	6,578(23.8)	7,744(28.0)	8,682(33.9)
Wet	15,694(25.7)	13,063(25.8)	10,399(23.9)	10,190(23.6)	9,865(24.2)	8,623(22.9)	7,773(24.2)	7,197(26.0)	7,747(28.0)	7,069(27.6)
Warm/cold therapy	27,377(44.9)	24,726(48.9)	21,553(49.6)	19,965(46.2)	18,852(46.3)	16,604(44.2)	16,439(51.3)	13,609(49.2)	14,312(51.7)	13,144(51.3)

Supplement Table S6. Annual medication prescription rate.

	2010	2011	2012	2013	2014	2015	2016	2017	2018	2019
	**N (%)**	**N (%)**	**N (%)**	**N (%)**	**N (%)**	**N (%)**	**N (%)**	**N (%)**	**N (%)**	**N (%)**
Total	79,948(43.7)	86,011(47.0)	95,968(47.6)	99,393(47.5)	99,637(47.6)	101,251(48.7)	104,042(50.4)	110,209(52.3)	112,761(52.2)	117,586(52.4)
Opioids	6,549(8.2)	14,240(16.6)	17,130(17.8)	19,407(19.5)	19,045(19.1)	19,741(19.5)	23,557(22.6)	29,623(26.9)	30,037(26.6)	32,170(27.4)
Non-Opioid Pain Relief Medication										
NSAIDS	47,778(59.8)	52,999(61.6)	59,842(62.4)	61,943(62.3)	62,551(62.8)	64,629(63.8)	69,221(66.5)	76,480(69.4)	78,897(70.0)	82,509(70.2)
Etc	1,672(2.1)	1,721(2.0)	2,181(2.3)	2,287(2.3)	2,253(2.3)	2,199(2.2)	6,501(6.2)	7,025(6.4)	7,868(7.0)	8,666(7.4)
Neuralgia medication	135(0.2)	175(0.2)	165(0.2)	159(0.2)	124(0.1)	78(0.1)	80(0.1)	148(0.1)	106(0.1)	141(0.1)
Muscle relaxants	26,951(33.7)	28,070(32.6)	31,142(32.5)	31,937(32.1)	30,952(31.1)	31,523(31.1)	33,788(32.5)	36,350(33.0)	38,064(33.8)	43,512(37.0)
Anesthetic	423(0.5)	546(0.6)	582(0.6)	483(0.5)	871(0.9)	1,162(1.1)	14,856(14.3)	36,647(33.3)	38,675(34.3)	41,889(35.6)
Gastrointestinal	41,431(51.8)	49,046(57.0)	60,844(63.4)	64,430(64.8)	64,233(64.5)	66,152(65.3)	68,361(65.7)	72,988(66.2)	74,840(66.4)	77,979(66.3)
Antipsychotic	7,038(8.8)	7,120(8.3)	6,842(7.1)	6,933(7.0)	6,545(6.6)	6,753(6.7)	6,197(6.0)	6,688(6.1)	6,167(5.5)	6,424(5.5)
Antibiotics										
Topical	5(0.0)	8(0.0)	8(0.0)	10(0.0)	1(0.0)	13(0.0)	109(0.1)	199(0.2)	201(0.2)	235(0.2)
Systemic	2,483(3.1)	2,776(3.2)	3,159(3.3)	3,386(3.4)	3,410(3.4)	3,465(3.4)	3,436(3.3)	3,626(3.3)	3,736(3.3)	3,704(3.2)
Steroids										
Topical	-	-	-	-	-	-	137(0.1)	321(0.3)	369(0.3)	350(0.3)
Systemic	2,094(2.6)	2,696(3.1)	3,112(3.2)	3,828(3.9)	3,569(3.6)	4,097(4.0)	13,608(13.1)	25,991(23.6)	28,213(25.0)	29,847(25.4)
Others	70,238(87.9)	72,317(84.1)	77,719(81.0)	80,019(80.5)	80,290(80.6)	81,580(80.6)	68,244(65.6)	54,160(49.1)	55,083(48.8)	41,346(35.2)

Supplement Table S7. Annual surgical treatment prescription rate.

	2010	2011	2012	2013	2014	2015	2016	2017	2018	2019
	**N (%)**	**N (%)**	**N (%)**	**N (%)**	**N (%)**	**N (%)**	**N (%)**	**N (%)**	**N (%)**	**N (%)**
Total	718(0.4)	980(0.5)	1,217(0.6)	1,374(0.7)	1,412(0.7)	1,498(0.7)	1,483(0.7)	1,594(0.8)	1,638(0.8)	1,721(0.8)
Acromioplasty and Repair of Ruptured Shoulder Rotator Cuff	652(90.8)	920(93.9)	1,163(95.6)	1,284(93.4)	1,334(94.5)	1,419(94.7)	1,384(93.3)	1,465(91.9)	1,491(91.0)	1,513(87.9)
Reconstruction of Tendon and Ligament	51(7.1)	58(5.9)	63(5.2)	77(5.6)	73(5.2)	85(5.7)	73(4.9)	95(6.0)	105(6.4)	88(5.1)
Excision of Joint, Shoulder	33(4.6)	36(3.7)	35(2.9)	43(3.1)	49(3.5)	53(3.5)	60(4.0)	101(6.3)	120(7.3)	135(7.8)
Excision of Joint, Others	-	2(0.2)	5(0.4)	2(0.1)	3(0.2)	4(0.3)	2(0.1)	5(0.3)	10(0.6)	6(0.3)
Replacement Arthroplasty , Shoulder	4(0.6)	8(0.8)	12(1.0)	20(1.5)	11(0.8)	22(1.5)	33(2.2)	29(1.8)	27(1.6)	52(3.0)
Removal of Implant for Internal Fixation	3(0.4)	4(0.4)	4(0.3)	10(0.7)	8(0.6)	1(0.1)	9(0.6)	6(0.4)	8(0.5)	7(0.4)
Open Reduction of Dislocation	-	-	-	-	-	-	-	1(0.1)	1(0.1)	-
Others	67(9.3)	63(6.4)	58(4.8)	74(5.4)	77(5.5)	90(6.0)	72(4.9)	74(4.6)	59(3.6)	98(5.7)

Supplement Table S8. Annual surgery rate for rotator cuff syndrome or tear (M751, S460).

	2010	2011	2012	2013	2014	2015	2016	2017	2018	2019
	**N (%)**	**N (%)**	**N (%)**	**N (%)**	**N (%)**	**N (%)**	**N (%)**	**N (%)**	**N (%)**	**N (%)**
Total	554(1.3)	722(1.4)	908(1.4)	1,054(1.5)	1,033(1.4)	1,135(1.5)	1,158(1.5)	1,286(1.5)	1,314(1.5)	1,409(1.5)
Acromioplasty and Repair of Ruptured Shoulder Rotator Cuff	527(95.1)	698(96.7)	873(96.1)	1,007(95.5)	1,002(97.0)	1,087(95.8)	1,093(94.4)	1,206(93.8)	1,238(94.2)	1,281(90.9)
Reconstruction of Tendon and Ligament	40(7.2)	43(6.0)	52(5.7)	67(6.4)	59(5.7)	67(5.9)	65(5.6)	79(6.1)	83(6.3)	76(5.4)
Excision of Joint, Shoulder	16(2.9)	22(3.0)	22(2.4)	19(1.8)	25(2.4)	27(2.4)	35(3.0)	59(4.6)	54(4.1)	69(4.9)
Excision of Joint, Others	-	1(0.1)	4(0.4)	1(0.1)	3(0.3)	2(0.2)	2(0.2)	5(0.4)	5(0.4)	5(0.4)
Replacement Arthroplasty , Shoulder	3(0.5)	8(1.1)	12(1.3)	20(1.9)	11(1.1)	20(1.8)	33(2.8)	29(2.3)	27(2.1)	52(3.7)
Removal of Implant for Internal Fixation	3(0.5)	1(0.1)	3(0.3)	8(0.8)	5(0.5)	1(0.1)	5(0.4)	5(0.4)	6(0.5)	5(0.4)
Open Reduction of Dislocation	-	-	-	-	-	-	-	1(0.1)	1(0.1)	-
Others	31(5.6)	36(5.0)	42(4.6)	45(4.3)	39(3.8)	53(4.7)	46(4.0)	49(3.8)	35(2.7)	68(4.8)

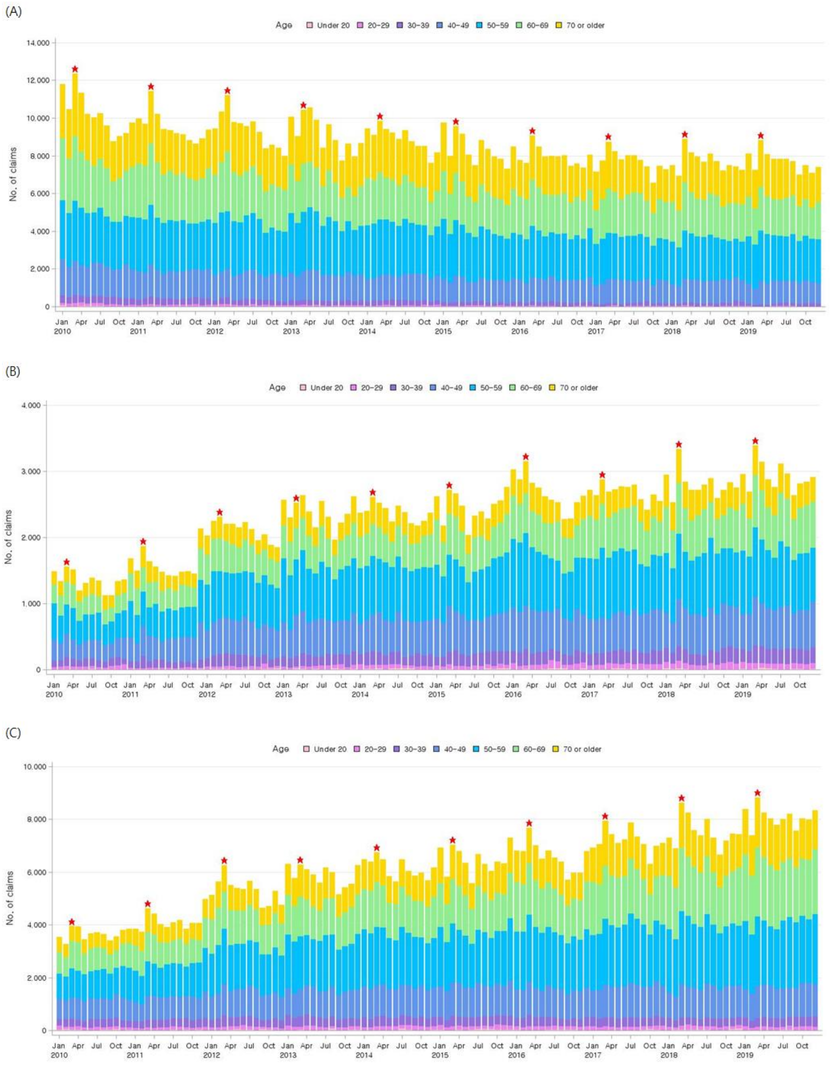

Figure S1. Ten-year (2010–2019) trend on monthly claims of medical services for shoulder disorders by age group. (A) adhesive capsulitis, (B) impingement syndrome, (C) rotator cuff syndrome or tear. Asterisk means March in each year.
